# Gastrointestinal Bleeding in COVID-19 Patients: A Systematic Review with Meta-Analysis

**DOI:** 10.1155/2021/2534975

**Published:** 2021-09-01

**Authors:** Giovanni Marasco, Marcello Maida, Gaetano Cristian Morreale, Massimo Licata, Matteo Renzulli, Cesare Cremon, Vincenzo Stanghellini, Giovanni Barbara

**Affiliations:** ^1^IRCCS Azienda Ospedaliero-Universitaria di Bologna, Bologna, Italy; ^2^Department of Medical and Surgical Science, University of Bologna, Bologna, Italy; ^3^Gastroenterology and Endoscopy Unit, S. Elia-Raimondi Hospital, Caltanissetta, Italy

## Abstract

The novel coronavirus disease 2019 (COVID-19) has been reported to affect the gastrointestinal system with a variety of symptoms, including bleeding. The prevalence of bleeding in these patients remains unclear. The aim of this meta-analysis is to estimate the rate of gastrointestinal bleeding in COVID-19 patients and its association with mortality. MEDLINE and Embase were searched through December 20, 2020. Studies reporting COVID-19 patients with and without gastrointestinal bleeding were included. Estimated prevalence with 95% confidence intervals (CI) was pooled; heterogeneity was expressed as *I*^2^. Metaregression analysis was performed to assess the impact of confounding covariates. Ten studies met the inclusion criteria and were included in the analysis. A total of 91887 COVID-19 patients were considered, of whom 534 reported gastrointestinal bleeding (0.6%) [409 (76.6%) upper and 121 (22.7%) lower gastrointestinal bleeding (UGIB and LGIB, resp.)]. The overall pooled gastrointestinal bleeding rate was 5% [95% CI 2–8], with high heterogeneity (*I*^2^ 99.2%); “small study effect” was observed using the Egger test (*p*=0.049). After removing two outlier studies, the pooled bleeding rate was 2% [95% CI 0–4], with high heterogeneity (*I*^2^ 99.2%), and no “small study effect” (*p*=0.257). The pooled UGIB rate was 1% (95% CI 0–3, *I*^2^ 98.6%, *p*=0.214), whereas the pooled LGIB rate was 1% (95% CI 0–2, *I*^2^ 64.7%, *p*=0.919). Metaregression analysis showed that overall estimates on gastrointestinal bleeding were affected by studies reporting different sources of bleeding. No significant association between gastrointestinal bleeding and mortality was found. In this meta-analysis of published studies, individuals with COVID-19 were found to be at risk for gastrointestinal bleeding, especially upper gastrointestinal bleeding.

## 1. Introduction

The novel coronavirus disease 2019 (COVID-19) initially described at the beginning of December 2019 in Wuhan, Hubei province of China, has evolved into a global pandemic [1]. The clinical course of severe acute respiratory syndrome coronavirus 2 (SARS-CoV-2) infection ranges from asymptomatic to a rapidly progressing and life-threatening disease most commonly associated with a variety of systemic and respiratory symptoms, such as fever, cough, dyspnoea, pneumonia, acute respiratory distress syndrome, systemic inflammatory response syndrome, and multiple organ failure [2]. Similar to other coronaviruses, SARS-CoV-2 infects the gastrointestinal tract [3, 4]. Alongside common gastrointestinal symptoms such as diarrhea, nausea, and vomiting [1], several case reports have described the occurrence of gastrointestinal bleeding in COVID-19 patients [5–9]. Gastrointestinal bleeding has been reported in a variable proportion, ranging from 2% to 13% in hospitalized patients [10–13]. A high prevalence of peptic ulcer disease complicated by bleeding was noticed in patients with moderate-to-severe acute respiratory distress syndrome caused by COVID-19 [14, 15]. However, the real burden of gastrointestinal bleeding in COVID-19 patients still needs to be clarified. Thus, we aimed to assess the prevalence of gastrointestinal bleeding in COVID-19 patients; in addition, we also evaluated the association between gastrointestinal bleeding and mortality in COVID-19 patients.

## 2. Materials and Methods

A systematic review and meta-analysis were carried out following the recommendations of the Cochrane Collaboration Group [16] and in line with PRISMA (Preferred Reporting Items for Systematic Reviews and Meta-Analyses) guidelines (16).

### 2.1. Information Sources and Search Strategies

Primary sources of the reviewed studies were MEDLINE via PubMed and Ovid Embase, which were searched systematically up to December 20, 2020.

Searches included combinations of the following medical subject headings (MeSH): “bleeding” or “hemorrhage” and “gastrointestinal” or “digestive” and “SARS-CoV-2” or “COVID-19.” The complete search strategies are reported in Supplementary material 1. The first report of cases of COVID-19 has been published on February 20, 2020 (17) which has been elected as the initial date for the literature search. The references list of the studies and relevant published reviews included were searched. There were no restrictions on language or publication status. Two authors (MM and GCM) carried out the initial selection based on titles and abstracts. A detailed full-text assessment of potentially relevant publications was independently carried out by the two reviewers, with any discrepancies being resolved through discussion or arbitration by a third reviewer (GM). Database searches were supplemented with literature searches of reference lists from potentially eligible articles to find additional studies.

### 2.2. Eligibility Criteria

Studies were selected for inclusion in the review if they met the following prespecified criteria: studies reporting cases of gastrointestinal bleeding within a cohort of COVID-19 patients. We used the following definitions for inclusion criteria: (1) upper gastrointestinal bleeding (UGIB) is defined as bleeding from a source proximal to the ligament of Treitz according to endoscopic or imaging findings [18]; (2) lower gastrointestinal bleeding (LGIB) refers to blood loss of recent onset originating from a site distal to the ligament of Treitz according to endoscopic or imaging findings [19]; (3) confirmed COVID-19 cases refer to the definitions according to the World Health Organization document released in March 2020 [20]; thus, a confirmed COVID-19 case is a person with laboratory confirmation of COVID-19 infection, irrespective of clinical signs and symptoms. Studies without a control group (represented by COVID-19 patients without gastrointestinal bleeding) and case reports were excluded. Studies that did not meet the inclusion criteria or in which essential information was missing or cannot be obtained from the authors were also excluded.

### 2.3. Data Collection Process and Quality Assessment

Relevant data were independently extracted by two authors (MM and GCM), using a standardized form. The following items were extracted from each study: year of publication, country, the total number of patients, including age and gender of the participants, the total number of COVID-19 patients with and without gastrointestinal bleeding, the rate of chronic use of anticoagulants and nonsteroidal anti-inflammatory drugs, and the total number of deaths in COVID-19 patients with and without gastrointestinal bleeding. In case of multiple publications for a single study, the latest publication was considered and supplemented, if necessary, with data from the previous publications. Two authors (MM and GCM) independently assessed the methodological quality of the included studies using the National Institute for Health and Care Excellence (NICE)-Quality Assessment of Diagnostic Accuracy Studies tool [21]. Discrepancies between reviewers concerning qualitative assessment were infrequent (overall interobserver variation <10%), and disagreements were resolved through discussion.

### 2.4. Statistical Analysis

The primary outcome was the pooled gastrointestinal bleeding rate. Secondary outcomes included the pooled UGIB and LGIB rates and the pooled mortality rate between groups with and without gastrointestinal bleeding. Rates of events were expressed as proportions for all studies and used to calculate the pooled gastrointestinal bleeding rate, pooled UGIB and LGIB rate, and pooled mortality rate. After data extraction, 95% confidence intervals (CI) of bleeding rates for each study were calculated using a random-effect model. Heterogeneity across the studies was assessed using the *I*^2^ statistic. In particular, the value of *I*^2^ describes the percentage of variability in point estimates which is due to heterogeneity rather than to sampling error: for an *I*^2^ < 50%, the risk of heterogeneity between studies was ranked low-moderate, whereas for *I*^2^ ≥ 50%, the risk of heterogeneity was ranked high [22]. We conducted sensitivity analyses excluding outliers, which were defined as studies in which the pooled estimates' (ES's) 95% CI was outside the 95% CI of the pooled ES (on both sides) and, excluding studies with a small number of participants, using an arbitrary cut-off of 100 patients. Indeed, calculations might differ from study to study, and larger studies provide more precise estimations [23]. All subsequent analyses have been performed using the sensitivity analysis which included the larger number of studies selected. Publication bias was investigated using the Egger test; a *p* value < 0.05 indicated a significant small size study effect. As part of the sensitivity analysis, the impact of confounding covariates [country, mean age of participants, sex, number of patients taking anticoagulants or nonsteroidal anti-inflammatory agents (NSAIDs), source of bleeding, and methodological quality of the studies included according to NICE] on the meta-analytic results was evaluated using metaregression analysis [24], reporting *β* coefficient ± standard error (SE). Since a low number of studies have been found, the *p* values were also recalculated using Monte Carlo permutation [25], with a number of 5000 permutations in order to obtain sufficient precision [26]. For assessing the mortality among patients with and without GI bleeding, odds ratio (OR) was calculated for each individual study; after, estimates were pooled, and 95% CI and *p* values were calculated. All analyses were carried out using STATA statistical software (Stata Corp., College Station, TX, USA).

## 3. Results

### 3.1. Study Selection

**Figure 1:**
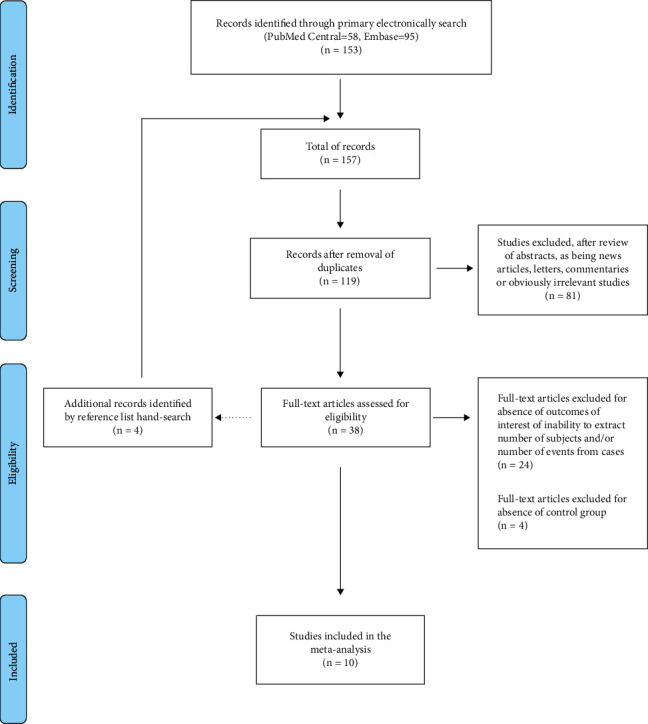
Preferred reporting items for systematic reviews and meta-analyses (PRISMA) flow diagram of the systematic literature search and studies included in the meta-analysis.

The electronic search identified a total of 158 records; after duplicates were removed, 119 articles were screened and finally, 38 full-text articles were assessed for eligibility (Figure 1). Of the 38 records selected, 24 were excluded for the absence of outcomes of interest or inability to extract the number of subjects and/or the number of events from cases and 4 were excluded for the absence of a control group.

Thus, a total of 10 studies [9–11, 15, 27–32], all full texts, met the eligibility criteria and were included in the meta-analysis.

### 3.2. Study Description

Table 1 resumes the main characteristics and the main outcomes of the studies included in the meta-analysis. Among the ten studies included, a total of 1481235 patients were considered, of whom 91887 (6.2%) were COVID-19 patients. Three studies were carried out in Europe (two in Italy [15, 28] and one in Spain [32]), three in the United State of America (USA) [9, 27, 31], three in China [10, 11, 30], and one in Israel [29]. All studies had a retrospective design. All studies reported only on COVID-19 patients except for two [27, 32]. Among COVID-19 patients, 534 reported gastrointestinal bleeding (0.6%), of whom 409 (76.6%) experienced UGIB and 121 (22.7%) LGIB. The rate of males included in the studies ranged from 40% [30] to 65.9% [9]. The mean age of patients included ranged from 45.3 [standard deviation (SD) 18.3] years [10] to 77 (SD 15) years [32]. Three studies [9, 28, 29] reported the rate of patients taking anticoagulants or antiplatelet drugs within all patients included, ranging from 37.4% [9] to 100% [28]. One study [15] reported only the number of patients taking these drugs among patients experiencing gastrointestinal bleeding. The most frequent gastrointestinal bleeding diagnosis was the presence of gastroduodenal ulcers [9, 15, 31, 32], followed by gastroduodenitis [10, 15, 28] and esophageal varices [11].

### 3.3. Quality Assessment

Table 2 summarizes the methodological quality evaluation of the studies included. All the studies showed high quality according to the NICE quality assessment scale, defined by a total score ≥4. Among the included studies, the lowest quality score of 4 was reported for three studies [10, 11, 30], mainly due to the lack of specifications on the inclusion and exclusion criteria and the absence of consecutive patients enrolment.

### 3.4. Pooled Bleeding Rate in COVID-19 Patients

**Figure 2:**
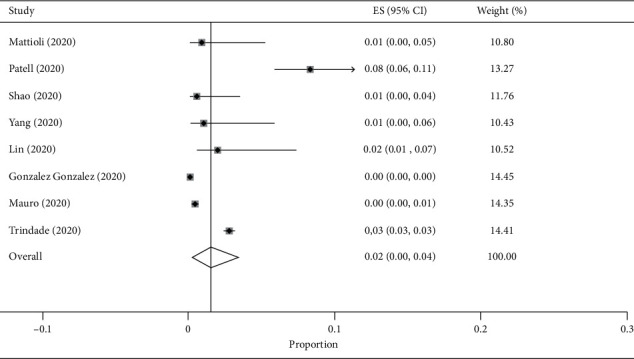
Forest plot of the pooled gastrointestinal bleeding rate in COVID-19 patients after removing 2 outlier's studies. ES: estimated proportion/prevalence; CI: confidence interval.

**Figure 3:**
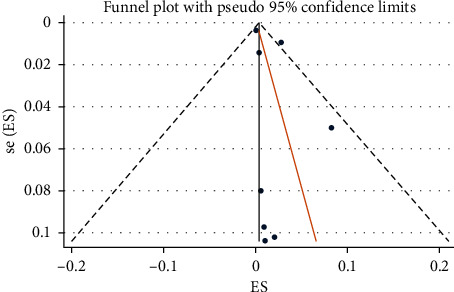
Funnel plot visual to asymmetry due to the “small sample size” effect after removing outlier's studies. SE of ES: standard error of estimated proportion/prevalence; ES: estimated proportion/prevalence; dotted black line: the line of pseudo 95% confidence limits; solid black line: the line of overall effect; and blue point: each study included.

The overall pooled gastrointestinal bleeding rate was 5% [95% CI: 2% to 8%], with high heterogeneity (*I*^2^ 99.2%); “small study effect” was observed using Egger test (*p*=0.049) (Supplementary Material 2 and 3). After removing two outliers' studies [9, 27], the pooled bleeding rate was 2% [95% CI: 0% to 4%], with high heterogeneity (*I*^2^ 99.2%), and no “small study effect” (*p*=0.257) (Figure 2). A visual assessment of funnel plot together with the Egger's test for publication bias after removing outliers' studies is reported in Figure 3. As a complementary part of sensitivity analysis, after removing three studies [10, 11, 27] reporting small cohorts, the pooled bleeding rate was 3% [95% CI: 1% to 7%], with high heterogeneity (*I*^2^ 99.4%), and no “small study effect” (*p*=0.112) (Supplementary Material 4). On the same line, after removing two outliers' studies [9, 27], the pooled UGIB rate was 1% (95% CI: 0% to 3%, *I*^2^ 98.6%, Egger's test *p*=0.214), whereas the pooled LGIB rate was 1% (95% CI: 0% to 2%, *I*^2^ 64.7%, Egger's test *p*=0.919) (Supplementary Materials 5 and 6, resp.).

Univariate metaregression analysis was used to explain potential sources of heterogeneity among the studies. Among the variables assessed, studies reporting different sources of bleeding (both UGIB and LGIB, *β* 1.029 ± 0.010, *p*=0.028) were the only variable explaining the high heterogeneity found for defining the overall gastrointestinal bleeding rate (Table 3). A borderline statistical significance was found for the geographical area of the study (*β* 0.978 ± 0.009, *p*=0.055), with higher heterogeneity associated with studies carried out in Eastern countries [10, 11, 30]. The quality of the studies did not affect our estimations (*β* 1.002 ± 0.020, *p*=0.909). After 5000 permutations were carried out, only studies reporting different sources of bleeding (both UGIB and LGIB, *p* = 0.037 ± 0.0027) remained associated with high heterogeneity (Table 3).

### 3.5. Bleeding and Mortality

Five studies [9–11, 28, 29] reported data on mortality in COVID-19 patients with and without gastrointestinal bleeding. After excluding three studies [10, 11, 28] reporting no deaths among patients with and without gastrointestinal bleeding, two studies were selected for this analysis [9, 29]. Overall, 2 out of 64 (3.1%) patients with gastrointestinal bleeding and 91 out of 457 (19.9%) patients without gastrointestinal bleeding were dead. The pooled OR for mortality between the two groups (gastrointestinal bleeding vs. no gastrointestinal bleeding) was 0.170 (95% CI 0.040 to 0.723, *p* 0.528), without heterogeneity between the two studies (*I*^2^ 0%) (Supplementary Material 7).

## 4. Discussion

This systematic review and meta-analysis of aggregate data from 10 studies assessing the rate of gastrointestinal bleeding in COVID-19 patients, showed an overall bleeding rate of 2%, of which 1% for UGIB and 1% for LGIB, respectively. Gastrointestinal bleeding is a common problem in the general population and in hospitalized patients, with reported rates for UGIB of 100–200 per 100000 persons annually and for LGIB of 20.5–27.0 per 100000 persons annually [33]. To date, only a few studies [27, 32] reported data on the comparison between gastrointestinal bleeding rates in patients with and without COVID-19, thus making not possible an accurate comparison of the bleeding risk between these two categories. However, considering the pooled gastrointestinal bleeding rates of COVID-19 patients as compared with the reported data of the general population, it is possible to speculate that COVID-19 patients have an increased risk of gastrointestinal bleeding. In addition, our results highlight that the potential source of variability of pooled gastrointestinal bleeding rates is the inclusion of studies reporting both UGIB and LGIB instead of only UGIB. Thus, it seems reasonable to assert that LGIB rates seem to confer high heterogeneity.

Only one large study [32] performed in 62 Spanish Emergency Departments (EDs) has compared the incidence of gastrointestinal bleeding in patients with or without COVID-19, showing a lower incidence of this event in patients with COVID-19 as compared with patients without COVID-19 (1.11% vs. 1.78%). However, in this study [32], COVID-19 patients underwent fewer endoscopies as compared with the control group, maybe due to infective related risks, thus leading to an underestimation of the real burden of gastrointestinal bleeding; moreover, another reason for gastrointestinal bleeding underestimation in this study is that most bleeding cases may occur during hospitalization, thus not recorded at EDs admission.

On the other hand, several other studies [9–11, 15, 27–31] suggested an increased gastrointestinal bleeding risk in COVID-19 patients. Among the potential mechanisms involved in bleeding, some authors hypothesized the development of an inflammation-induced coagulopathy and thromboinflammation, in addition to the direct damage of the virus on the gastrointestinal mucosa [34]. Indeed, SARS-CoV-2 is able to infect enteric cells since the highest expression of angiotensin-converting enzyme 2 in the human body, which is the viral binding site, occurs in the brush border of intestinal enterocytes [35, 36]. Moreover, SARS-CoV-2 nucleocapsid proteins have been detected in the cytoplasm of gastric, duodenal, and rectal cells of COVID-19 patients with SARS-Cov-2 fecal shedding [12]. A direct consequence of SARS-CoV-2 infection is inflammation of gastrointestinal mucosa [12] and the reduction of the epithelial cell functional mass [37]. Since bleeding occurred mainly during hospitalization, a multifactorial etiology has been postulated. Indeed, most patients admitted to the hospital were given thromboprophylaxis and other treatments, which may represent an additional risk factor for bleeding. Given the emerging evidence of thrombotic risks in COVID-19 patients [38], it should be underlined that most of the symptomatic COVID-19 patients took anticoagulants, such as heparin, at least at prophylactic doses, for preventing COVID-19 related prothrombotic activity. Heparin treatment has been recently reported to be an effective therapy [39]. Gastrointestinal bleeding may also be explained by COVID-19-associated coagulopathy inducing increased D-dimer and fibrinogen levels, which may predispose to a high risk of thrombosis, thus explaining the occurrence of ischemic colitis [40]. Other putative mechanisms underlying gastrointestinal bleeding may be represented by ulcers occurring under conditions of severe stress such as hospitalization [8, 15] or disseminated intravascular coagulation, which is a hypercoagulable state also inducing bleeding.

Based on only two studies [9, 29] reporting on the survival of COVID-19 patients with and without gastrointestinal bleeding, we additionally found that bleeding was a protective factor. We believe that this latter result is strongly biased by the rather low number of included studies. Similar to our results, discordant data are available up to now on this topic [31, 32].

To our knowledge, this is the first meta-analysis aimed at assessing the pooled rate of gastrointestinal bleeding in COVID-19 patients. Our meta-analysis has several strengths. First, a comprehensive literature search minimizes the risk of missing studies. Moreover, the presence of a large cohort of patients from different countries makes our reported rates globally generalizable. In addition, all studies included were ranked as having a high quality according to the NICE quality assessment scale. Another strength of our meta-analysis is the sensitivity analysis performed excluding those studies source of publication bias, thus obtaining more reliable results. Moreover, maybe due to the COVID-19 pandemic irruption in everyday clinical practice and research activity, influencing the study's design and publication process, we expected to find a high heterogeneity among included studies. As additional strength of our analysis, we performed a univariate metaregression analysis to find sources of heterogeneity. The only risk factor identified by this analysis was the presence of studies reporting sources of bleeding different from UGIB.

At the same time, this meta-analysis has some weaknesses. Among these, the small number of included studies could have led to an underestimation of the gastrointestinal bleeding pooled rates. Moreover, we considered studies reporting on COVID-19 patients from different settings (e.g., inpatients [15], records from endoscopy units [27], or EDs [32]), thus with different degrees of COVID-19 clinical course, which may have influenced the reported pooled estimates. Nevertheless, it was not possible to extract exact numbers of events from each of these specific categories both in cases and in control groups, so to perform subgroup analysis. In addition, since only a few included studies reported on UGIB and LGIB confirmed by endoscopy, we considered the number of UGIB and LGIB as reported by each author even without the presence of a confirmatory endoscopy, thus leading to a misclassification bias for the definition of patients with and without gastrointestinal bleeding. Moreover, in our meta-analysis, it was not possible to consider other predisposing conditions for gastrointestinal bleeding. Finally, only a few studies [9, 28, 29] reported on the use of anticoagulants and NSAIDs, treatments that may influence bleeding rates. However, considering only these three studies, our metaregression analysis showed that this factor did not influence our pooled results, confirming previous results on an absent influence of anticoagulation on gastrointestinal bleeding occurrence [9].

In conclusion, our results provide evidence that patients with COVID-19 present a gastrointestinal bleeding risk of 2%. We are still unable to clearly state whether these patients have an increased risk of gastrointestinal bleeding when compared to patients without COVID-19. Our data should be considered by clinicians when managing COVID-19 patients at gastrointestinal bleeding risk for other conditions or before starting anticoagulants. As included studies were conducted in centers from different countries all over the world, we believe our findings are generalizable worldwide. Recently, effective anti-COVID-19 vaccines have been developed [41, 42], and distribution is ongoing. However, full vaccination coverage will require months; in this view, further well-designed studies evaluating the real risk of gastrointestinal bleeding in COVID-19 patients and its pathophysiology will be still useful in clinical practice.

## Figures and Tables

**Table 1:** Characteristics of the studies included in the meta-analysis.

Author	Year	Country	Patients included/COVID-19 patients, (*n*)	Sex (male), *n* (%)	Age [mean (SD) or median (IQR/range)]	Patients taking anticoagulants or NSAIDs (*n*)	Bleeding, *n* (%)	Most frequent diagnosis for GI bleeding
Blackett et al. [27]	2020	USA	545/79	328 (60.2)	63 (52–73)	—	35 (44.3)	—

Martin et al. [9]	2020	USA	123/123	81 (65.9)	68.7 (15.1)	46 (37.4)	41 (33.3)	Duodenal and rectal ulcers

Mattioli et al. [28]	2020	Italy	105/105	58 (55.2)	73.7 (14.6)	105 (100)	1 (1)	Erosive gastritis

Patell et al. [29]	2020	Israel	398/398	209 (52.5)	65	369 (92.7)	33 (.3)	—

Shao et al. [30]	2020	China	155/155	62 (40)	48 (33–63)	—	1 (0.6)	—

Yang et al. [11]	2020	China	92/92	49 (53.3)	69.8 (14.5)	—	1 (1.1)	Variceal bleeding

Lin et al. [10]	2020	China	95/95	45 (47.4)	45.3 (18.3)	—	2 (2.1)	Gastroduodenitis

Gonzalez Gonzalez et al. [32]	2020	Spain	1463693/74814	45935 (61.4)	77 (15)	—	83 (0.1)	Ulcers

Mauro et al. [15]	2020	Italy	4871/4871	—	75	22 (only among bleedings)	23 (0.5)	Peptic ulcer, haemorrhagic gastritis

Trindade et al. [31]	2020	USA	11158/11158	—	69.4 (14.3)	—	314 (2.8)	Gastroduodenal ulcers

COVID-19 = coronavirus 2019 associated disease; *n* = number; SD = standard deviation; IQR = interquartile range; GI = gastrointestinal; and USA = United States of America.

**Table 2:** Risk of bias and applicability concerns of the studies included in the meta-analysis.

Author	NICE quality assessment score-ITEMS								Total NICE score^*∗*^
	NICE 1: case series collected in more than one centre?	NICE 2: is the objective of the study clearly described?	NICE 3: are the inclusion and exclusion criteria clearly reported?	NICE 4: is there a clear definition of the outcome reported?	NICE 5: were data collected prospectively?	NICE 6: is there an explicit statement that the patients were recruited consecutively?	NICE 7: are the main findings of the study clearly described?	NICE 8: Are outcomes stratified?	
Blackett et al. [27]	Yes	Yes	No	Yes	No	No	Yes	Yes	**5**

Martin et al. [9]	Yes	Yes	No	Yes	No	No	Yes	Yes	**5**

Mattioli et al. [28]	No	Yes	Yes	Yes	No	Yes	Yes	Yes	**6**

Patell et al. [29]	No	Yes	Yes	Yes	No	Yes	Yes	Yes	**6**

Shao et al. [30]	No	Yes	No	Yes	No	No	Yes	Yes	**4**

Yang et al. [11]	No	Yes	No	Yes	No	No	Yes	Yes	**4**

Lin et al. [10]	No	Yes	No	Yes	No	No	Yes	Yes	**4**

Gonzalez Gonzalez et al. [32]	Yes	Yes	No	Yes	No	No	Yes	Yes	**5**

Mauro et al. [15]	Yes	Yes	Yes	Yes	No	No	Yes	Yes	**6**

Trindade et al. [31]	Yes	Yes	No	Yes	No	No	Yes	Yes	**5**

The quality of selected studies was independently assessed by two investigators (GM and MM) using the National Institute of Clinical Excellence (NICE) quality assessment scale (1). Higher-quality studies were defined by a total score ≥4 and “lower-quality studies” by a total score <4. Discrepancies between reviewers concerning qualitative assessment were infrequent (overall interobserver variation <10%).

**Table 3:** Results of univariable metaregression analysis performed after removing outlier's studies.

Covariates	Number of studies	Beta coefficient ± SE	Adjusted *R*^2^ (%)	*p* value	*p* value ± SE after Monte Carlo permutation
Country	8	0.978 (±0.009)	100	0.055	0.417 ± 0.0070
Age	6	0.998 (±0.002)	100	0.423	0.592 ± 0.0060
Sex (male)	6	0.999 (±7.54^−07^)	100	0.274	0.298 ± 0.0065
Anticoagulants/NSAIDs intake	3	1.000 (±0.0003)	—	0.589	0.339 ± 0.0067
Source of bleeding (UGIB vs. UGIB + LGIB)	8	1.029 (±0.010)	100	0.028	0.037 ± 0.0027
NICE quality assessment scale score	8	1.002 (±0.020)	−50.74	0.909	0.967 ± 0.0025

SE = standard error; *R*^2^ = relative reduction in between-study variance: the value indicates the proportion of between study variance explained by covariate; NSAIDs = nonsteroidal anti-inflammatory agents; UGIB = upper gastrointestinal bleeding; LGIB = lower gastrointestinal bleeding; and NICE = National Institute of Clinical Excellence.

## Data Availability

The data used to support the findings of this study are included within the article.

## Supplementary Materials

Supplementary Materials *Supplementary Material 1.* Complete electronic search strategies for studies included in the systematic review and metanalysis. *Supplementary Material 2*. Forest plot of the pooled gastrointestinal bleeding rate in COVID-19 patients. ES: estimated proportion/prevalence; CI: confidence interval. *Supplementary Material 3*. Funnel plot visual to asymmetry due to the “small sample size.” *Legend*. SE of ES: standard error of estimated proportion/prevalence; ES: estimated proportion/prevalence; dotted black line: the line of pseudo 95% confidence limits; solid black line: the line of overall effect; blue point: each study included. *Supplementary Material 4*. Forest plot of the pooled gastrointestinal bleeding rate in COVID-19 patients after removing small studies. ES: estimated proportion/prevalence; CI: confidence interval. *Supplementary Material 5*. Forest plot of the pooled upper gastrointestinal bleeding (UGIB) rate in COVID-19 patients after removing outlier's studies. ES: estimated proportion/prevalence; CI: confidence interval. *Supplementary Material 6*. Forest plot of the pooled lower gastrointestinal bleeding (LGIB) rate in COVID-19 patients after removing outlier's studies. ES: estimated proportion/prevalence; CI: confidence interval. *Supplementary Material 7*. Forest plot of the pooled mortality among COVID-19 patients with and without gastrointestinal bleeding. OR: odds ratio; CI: confidence interval.

## References

[1] Huang C., Wang Y., Li X. (2020). Clinical features of patients infected with 2019 novel coronavirus in Wuhan, China. *The Lancet*.

[2] Zhou F., Yu T., Du R. (2020). Clinical course and risk factors for mortality of adult inpatients with COVID-19 in Wuhan, China: a retrospective cohort study. *The Lancet*.

[3] Openshaw P. J. (2009). Crossing barriers: infections of the lung and the gut. *Mucosal Immunology*.

[4] Saif L. J. (2010). Bovine respiratory coronavirus. *Veterinary Clinics of North America: Food Animal Practice*.

[5] Carvalho A., Alqusairi R., Adams A. (2020). SARS-CoV-2 gastrointestinal infection causing hemorrhagic colitis: implications for detection and transmission of COVID-19 disease. *American Journal of Gastroenterology*.

[6] Tian Y., Rong L., Nian W., He Y. (2020). Review article: gastrointestinal features in COVID-19 and the possibility of faecal transmission. *Alimentary Pharmacology & Therapeutics*.

[7] Pan L., Mu M., Yang P. (2020). Clinical characteristics of COVID-19 patients with digestive symptoms in Hubei, China: a descriptive, cross-sectional, multicenter study. *American Journal of Gastroenterology*.

[8] Gadiparthi C., Perisetti A., Sayana H., Tharian B., Inamdar S., Korman A. (2020). Gastrointestinal bleeding in patients with severe SARS-CoV-2. *American Journal of Gastroenterology*.

[9] Martin T. A., Wan D. W., Hajifathalian K. (2020). Gastrointestinal bleeding in patients with coronavirus disease 2019: a matched case-control study. *American Journal of Gastroenterology*.

[10] Lin L., Jiang X., Zhang Z. (2020). Gastrointestinal symptoms of 95 cases with SARS-CoV-2 infection. *Gut*.

[11] Yang F., Shi S., Zhu J., Shi J., Dai K., Chen X. (2020). Analysis of 92 deceased patients with COVID‐19. *Journal of Medical Virology*.

[12] Xiao F., Tang M., Zheng X., Liu Y., Li X., Shan H. (2020). Evidence for gastrointestinal infection of SARS-CoV-2. *Gastroenterology*.

[13] Marasco G., Lenti M. V., Cremon C. (2021). Implications of SARS-CoV-2 infection for neurogastroenterology. *Neuro-Gastroenterology and Motility: The Official Journal of the European Gastrointestinal Motility Society*.

[14] Melazzini F., Lenti M. V., Mauro A., De Grazia F., Di Sabatino A. (2020). Peptic ulcer disease as a common cause of bleeding in patients with coronavirus disease 2019. *American Journal of Gastroenterology*.

[15] Mauro A., De Grazia F., Lenti M. V. (2020). Upper gastrointestinal bleeding in COVID-19 inpatients: incidence and management in a multicenter experience from Northern Italy. *Clinics and Research in Hepatology and Gastroenterology*.

[16] (2018). Handbook for dta reviews|cochrane screening and diagnostic tests n.d. http://methods.cochrane.org/sdt/handbook-dta-reviews.

[17] Zhu N., Zhang D., Wang W. (2020). A novel coronavirus from patients with pneumonia in China, 2019. *New England Journal of Medicine*.

[18] (2007). *Acute Upper Gastrointestinal Bleed. Most Common Inpatient Problems in Internal Medicine.*.

[19] Zuccaro G. (1998). Management of the adult patient with acute lower gastrointestinal bleeding. *American Journal of Gastroenterology*.

[20] (2020). Coronavirus disease (COVID-19) n.D. https://www.who.int/emergencies/diseases/novel-coronavirus-2019.

[21] National Institute of Clinical Excellence (NICE) (2020). Quality assessment scale. https://www.nice.org.uk/guidance/cg-3/documents/appendix-4-quality-of-case-series-form2.

[22] Higgins J. P. T., Thompson S. G. (2002). Quantifying heterogeneity in a meta-analysis. *Statistics in Medicine*.

[23] Inthout J., Ioannidis J. P. A., Borm G. F., Goeman J. J. (2015). Small studies are more heterogeneous than large ones: a meta-meta-analysis. *Journal of Clinical Epidemiology*.

[24] Thompson S. G., Sharp S. J. (1999). Explaining heterogeneity in meta-analysis: a comparison of methods. *Statistics in Medicine*.

[25] Higgins J. P. T., Thompson S. G. (2004). Controlling the risk of spurious findings from meta-regression. *Statistics in Medicine*.

[26] Manly B. F. J. (2018). *Randomization, Bootstrap and Monte Carlo Methods in Biology*.

[27] Blackett J. W., Kumta N. A., Dixon R. E. (2020). Characteristics and outcomes of patients undergoing endoscopy during the COVID-19 pandemic: a multicenter study from New York city. *Digestive Diseases and Sciences*.

[28] Mattioli M., Benfaremo D., Mancini M. (2020). Safety of intermediate dose of low molecular weight heparin in COVID-19 patients. *Journal of Thrombosis and Thrombolysis*.

[29] Patell R., Bogue T., Bindal P. (2020). Incidence of thrombosis and hemorrhage in hospitalized cancer patients with COVID‐19. *Journal of Thrombosis and Haemostasis*.

[30] Shao L., Li X., Zhou Y. (2020). Novel insights into illness progression and risk profiles for mortality in non-survivors of COVID-19. *Frontiers of Medicine*.

[31] Trindade A. J., Izard S., Coppa K. (2020). Gastrointestinal bleeding in hospitalized COVID‐19 patients: a propensity score matched cohort study. *Journal of Internal Medicine*.

[32] González González R., Jacob J., Miró Ò. (2020). Incidence, clinical characteristics, risk factors, and outcomes of upper gastrointestinal bleeding in patients with COVID-19. *Journal of Clinical Gastroenterology*.

[33] Farrell J. J., Friedman L. S. (2005). Review article: the management of lower gastrointestinal bleeding. *Alimentary Pharmacology and Therapeutics*.

[34] Gupta A., Madhavan M. V., Sehgal K. (2020). Extrapulmonary manifestations of COVID-19. *Nature Medicine*.

[35] (2020). ACE2 protein expression summary-the human protein atlas N. https://www.proteinatlas.org/ENSG00000130234-ACE2.

[36] Qi F., Qian S., Zhang S., Zhang Z. (2020). Single cell RNA sequencing of 13 human tissues identify cell types and receptors of human coronaviruses. *Biochemical and Biophysical Research Communications*.

[37] Uzzan M., Soudan D., Peoc’h K., Weiss E., Corcos O., Treton X. (2020). Patients with COVID-19 present with low plasma citrulline concentrations that associate with systemic inflammation and gastrointestinal symptoms. *Digestive and Liver Disease*.

[38] Tang N., Li D., Wang X., Sun Z. (2020). Abnormal coagulation parameters are associated with poor prognosis in patients with novel coronavirus pneumonia. *Journal of Thrombosis and Haemostasis*.

[39] Di Castelnuovo A. F., Costanzo S., Iacoviello L. (2021). Heparin in COVID-19 patients is associated with reduced in-hospital mortality: the multicentre Italian CORIST Study. *Thrombosis & Haemostasis*.

[40] Chan K. H., Lim S. L., Damati A. (2020). Coronavirus disease 2019 (COVID-19) and ischemic colitis: an under-recognized complication. *The American Journal of Emergency Medicine*.

[41] Jackson L. A., Anderson E. J., Rouphael N. G. (2020). An mRNA vaccine against SARS-CoV-2 - preliminary report. *New England Journal of Medicine*.

[42] Polack F. P., Thomas S. J., Kitchin N. (2020). Safety and efficacy of the BNT162b2 mRNA covid-19 vaccine. *New England Journal of Medicine*.

