# Supplementary material for: Gastrointestinal Bleeding in COVID-19 Patients: A Systematic Review with Meta-Analysis
Source: Can J Gastroenterol Hepatol. 2021 Sep 1;2021:2534975. doi: 10.1155/2021/2534975 (PMC8429023; doi:10.1155/2021/2534975)

**Supplementary material 1.** Complete electronic search strategies for studies included in the systematic review and metanalysis.

**MEDLINE via PUBMED**

((bleeding[TIAB]) OR (haemorrhage[TIAB]) OR (hemorrhage[TIAB])) AND ((gastrointestinal[TIAB]) OR (digestive[TIAB]) OR (duodenal[TIAB]) OR (duodenum[TIAB]) OR (stomach[TIAB]) OR (gastric[TIAB]) OR (oesophageal[TIAB]) OR (oesophagus[TIAB]) OR (small gut[TIAB]) OR (small intestine[TIAB]) OR (colonic[TIAB]) OR (colon[TIAB])) AND ((sars cov 2[TIAB]) OR (covid[TIAB]) OR (covid19[TIAB]) OR (covid-19[TIAB]))

**EMBASE**

((bleeding:ab,ti) OR (haemorrhage:ab,ti) OR (hemorrhage:ab,ti)) AND ((gastrointestinal:ab,ti) OR (digestive:ab,ti) OR (duodenal:ab,ti) OR (duodenum:ab,ti) OR (stomach:ab,ti) OR (gastric:ab,ti) OR (oesophageal:ab,ti) OR (oesophagus:ab,ti) OR (small gut:ab,ti) OR (small intestine:ab,ti) OR (colonic:ab,ti) OR (colon:ab,ti)) AND ((sars cov 2:ab,ti) OR (covid:ab,ti) OR (covid19:ab,ti) OR (covid-19:ab,ti))

**Supplementary material 2.** Forest plot of the pooled gastrointestinal bleeding rate in COVID-19 patients. ES: Estimated proportion/prevalence; CI: Confidence Interval.


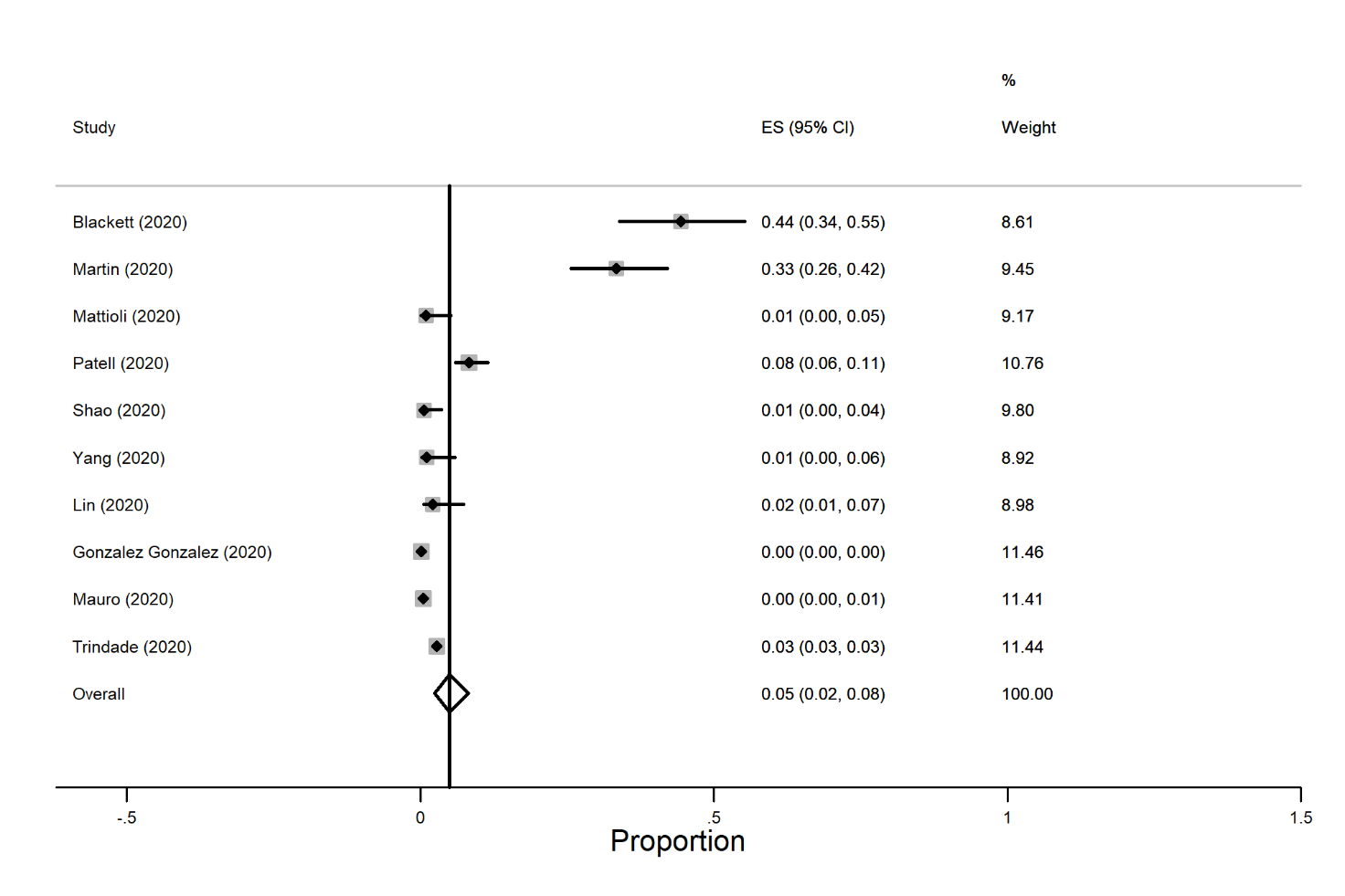


**Supplementary material 3.** Funnel plot visual to Asymmetry due to the “small sample size”. Legend: SE of ES: Standard Error of estimated proportion/prevalence; ES: Estimated proportion/prevalence; Dotted black line: the line of pseudo 95% confidence limits; Solid black line: the line of overall effect; Blue point: each study included.


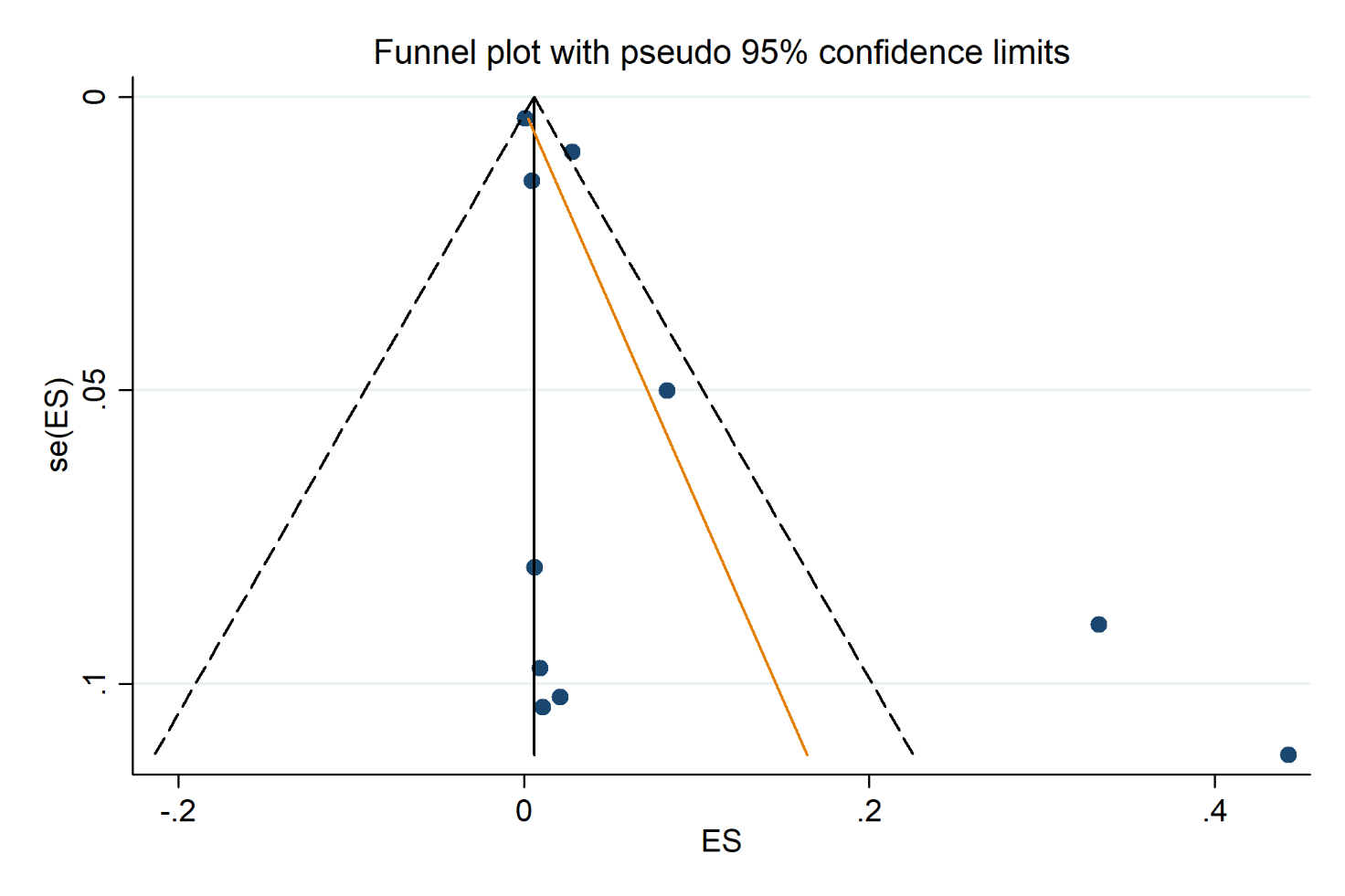


**Supplementary material 4.** Forest plot of the pooled gastrointestinal bleeding rate in COVID-19 patients after removing 3 small studies. ES: Estimated proportion/prevalence; CI: Confidence Interval.


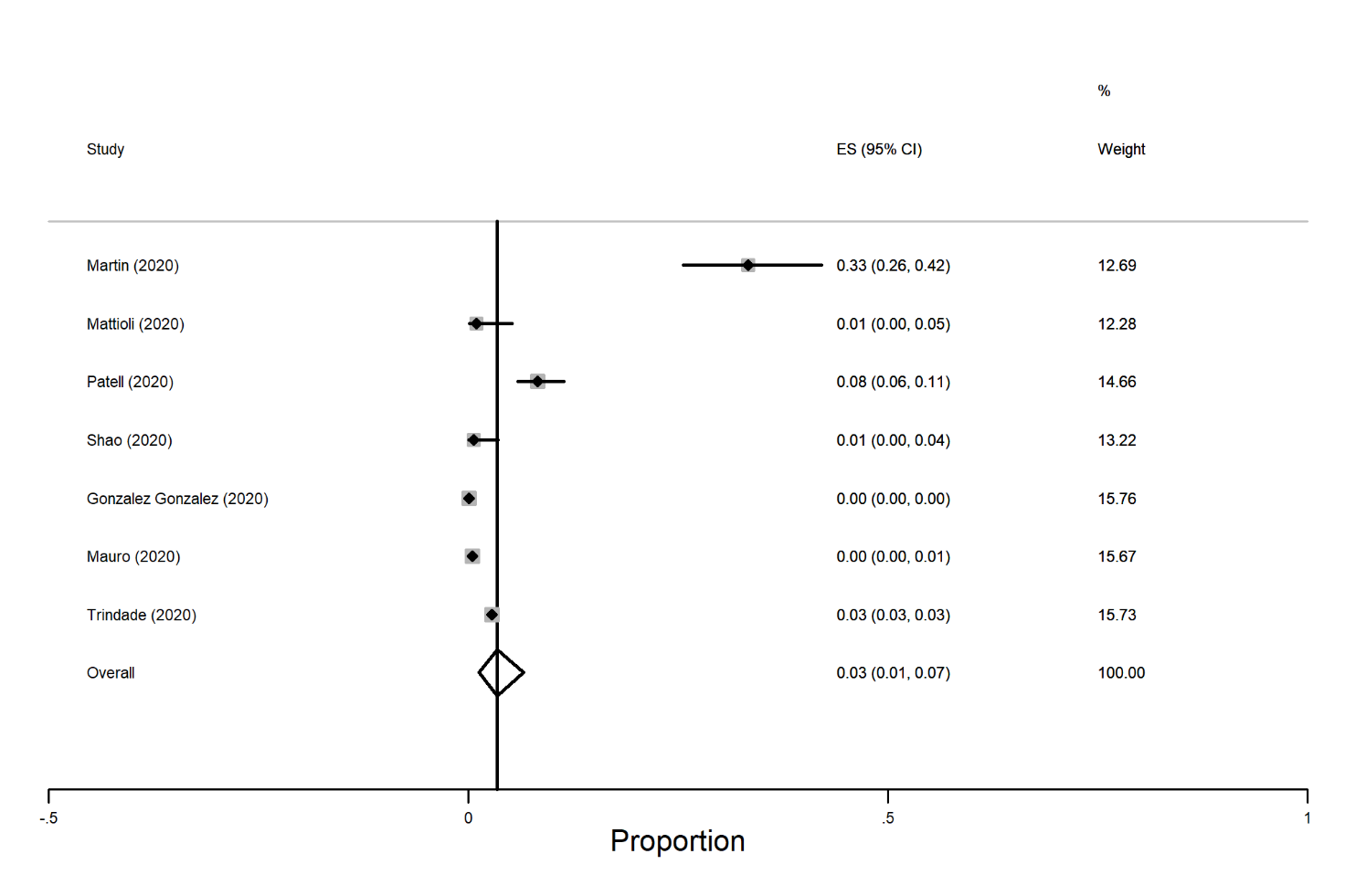


**Supplementary material 5.** Forest plot of the pooled upper gastrointestinal bleeding (UGIB) rate in COVID-19 patients after removing outlier’s studies. ES: Estimated proportion/prevalence; CI: Confidence Interval.


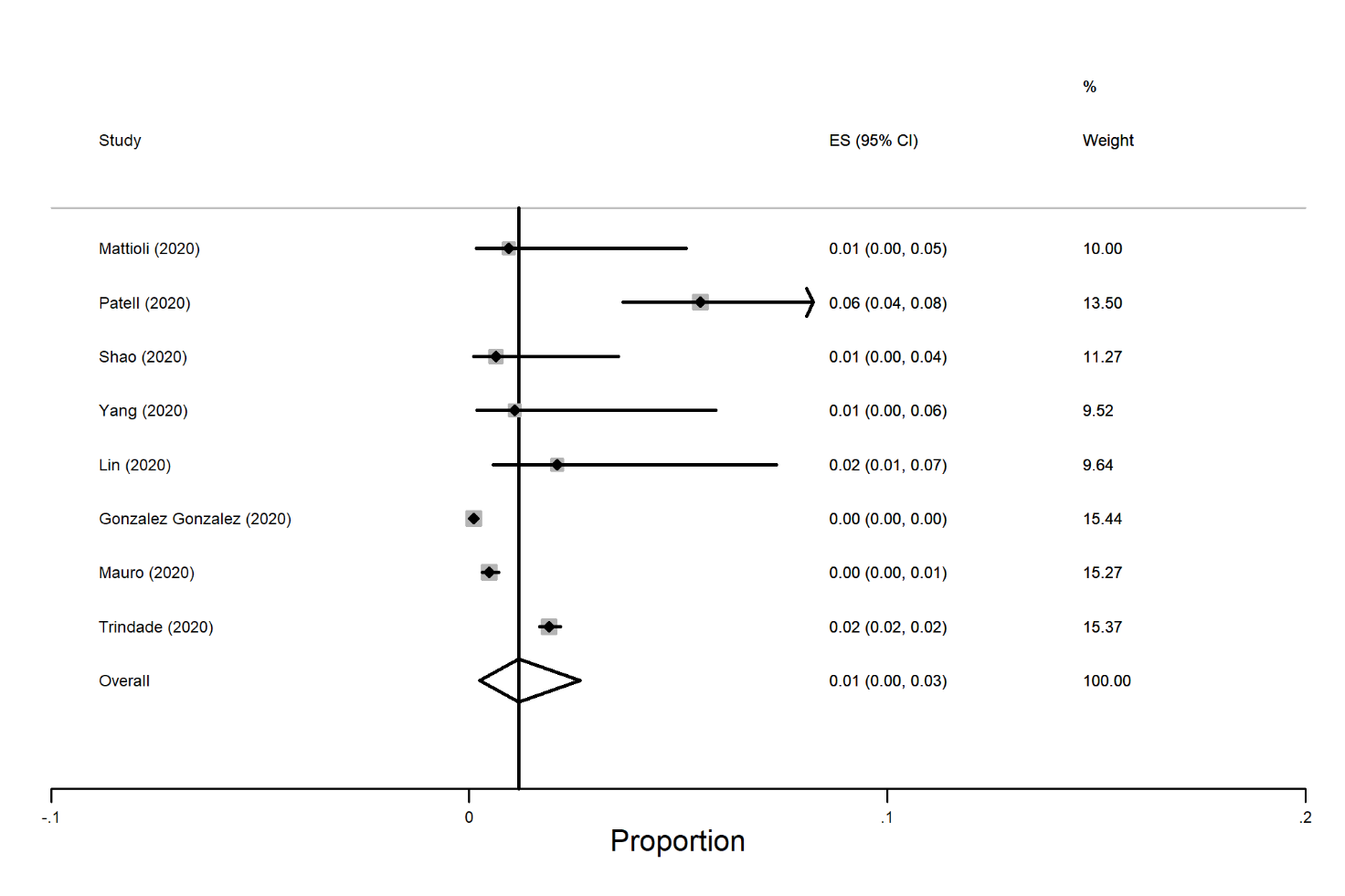


**Supplementary material 6.** Forest plot of the pooled lower gastrointestinal bleeding (LGIB) rate in COVID-19 patients after removing outlier’s studies. ES: Estimated proportion/prevalence; CI: Confidence Interval.


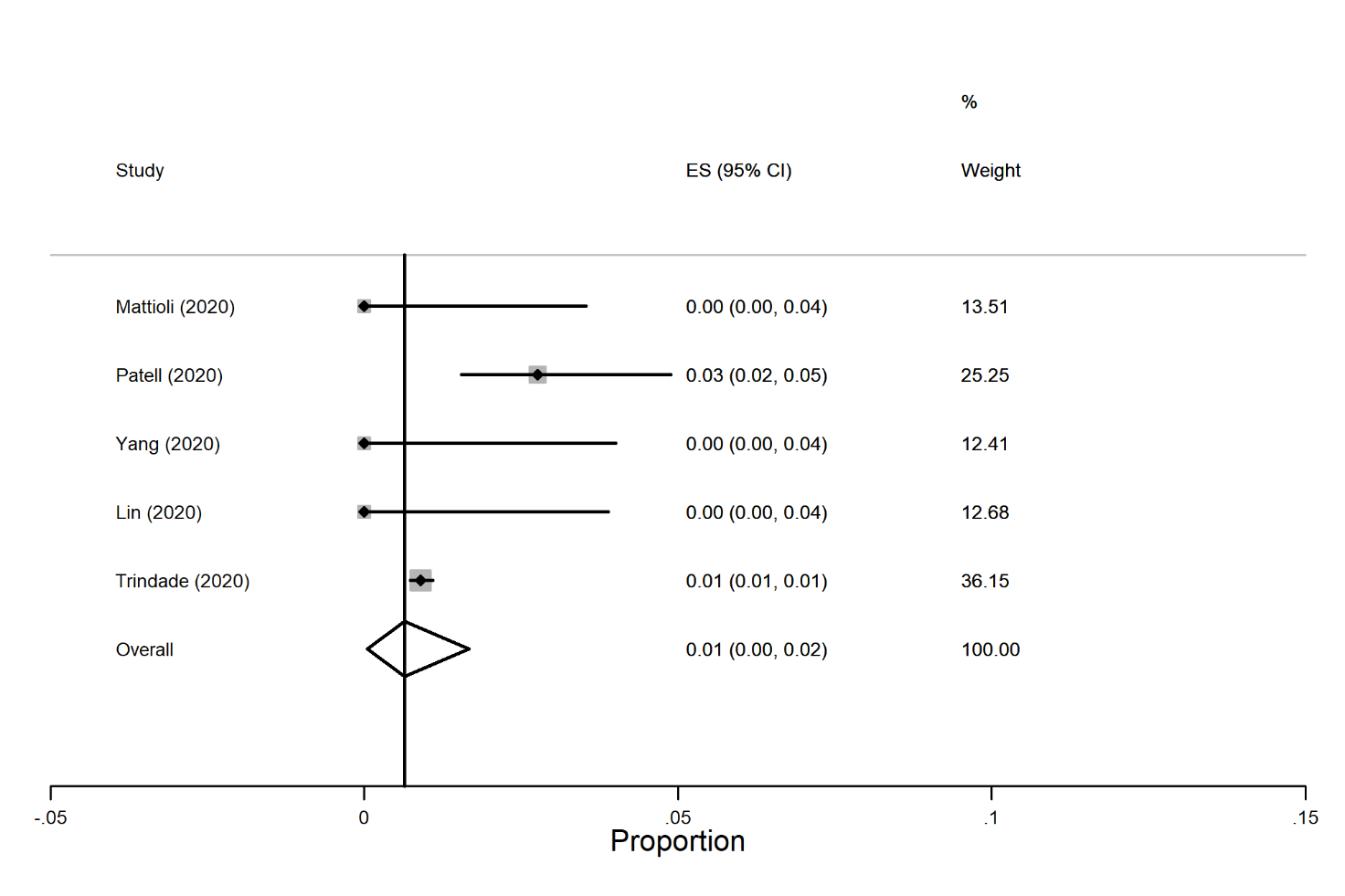


**Supplementary material 7.** Forest plot of the pooled mortality among COVID-19 patients with and without GI bleeding. OR: Odds ratio; CI: Confidence Interval.


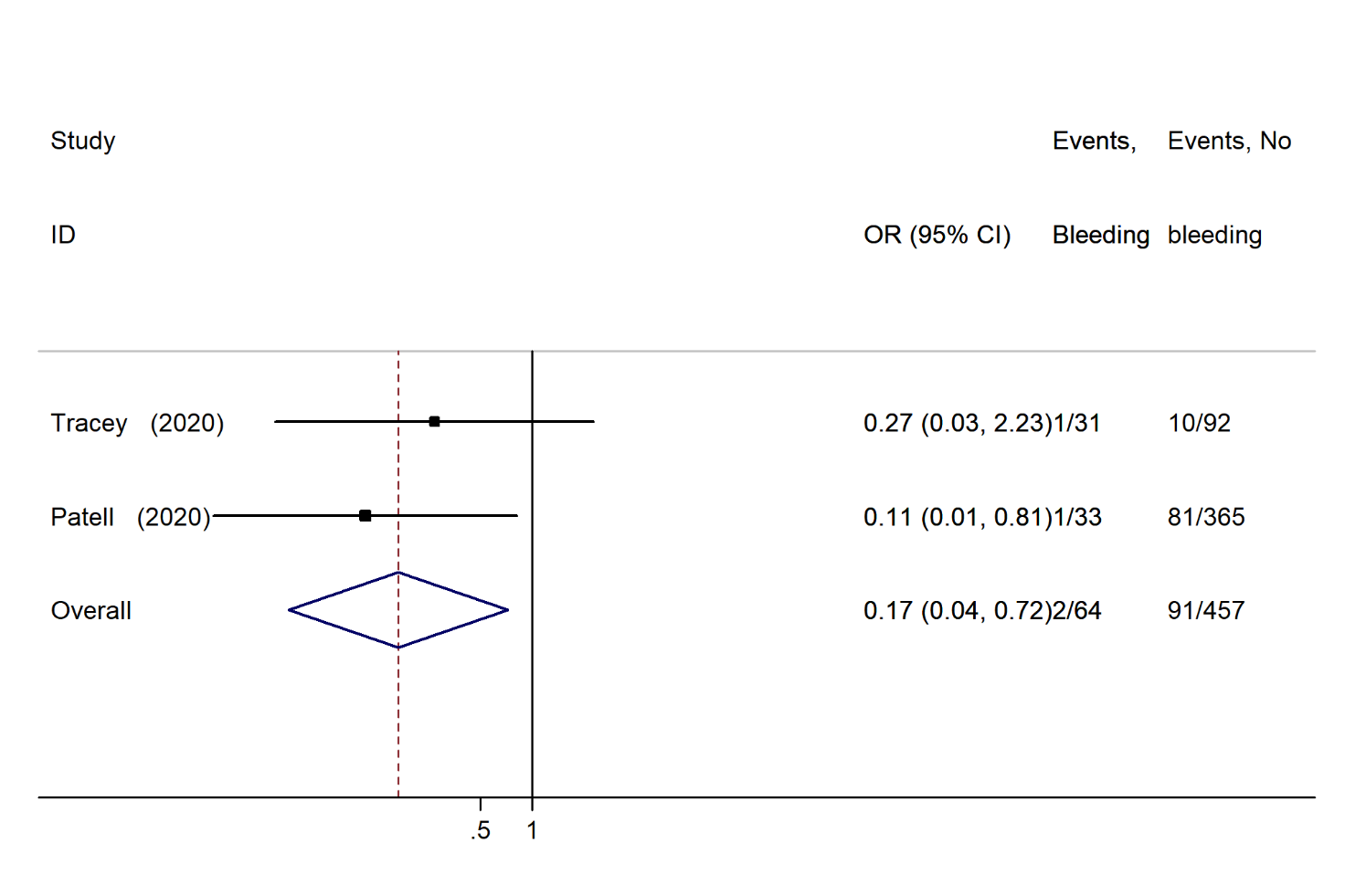

Supplement: Supplementary Materials — Supplementary Material 1. Complete electronic search strategies for studies included in the systematic review and metanalysis. Supplementary Material 2. Forest plot of the pooled gastrointestinal bleeding rate in COVID-19 patients. ES: estimated proportion/prevalence; CI: confidence interval. Supplementary Material 3. Funnel plot visual to asymmetry due to the “small sample size.” Legend. SE of ES: standard error of estimated proportion/prevalence; ES: estimated proportion/prevalence; dotted black line: the line of pseudo 95% confidence limits; solid black line: the line of overall effect; blue point: each study included. Supplementary Material 4. Forest plot of the pooled gastrointestinal bleeding rate in COVID-19 patients after removing small studies. ES: estimated proportion/prevalence; CI: confidence interval. Supplementary Material 5. Forest plot of the pooled upper gastrointestinal bleeding (UGIB) rate in COVID-19 patients after removing outlier's studies. ES: estimated proportion/prevalence; CI: confidence interval. Supplementary Material 6. Forest plot of the pooled lower gastrointestinal bleeding (LGIB) rate in COVID-19 patients after removing outlier's studies. ES: estimated proportion/prevalence; CI: confidence interval. Supplementary Material 7. Forest plot of the pooled mortality among COVID-19 patients with and without gastrointestinal bleeding. OR: odds ratio; CI: confidence interval. [file 2534975.f1.docx]
